# MicroRNA and Alternative mRNA Splicing Events in Cancer Drug Response/Resistance: Potent Therapeutic Targets

**DOI:** 10.3390/biomedicines9121818

**Published:** 2021-12-02

**Authors:** Rahaba Marima, Flavia Zita Francies, Rodney Hull, Thulo Molefi, Meryl Oyomno, Richard Khanyile, Sikhumbuzo Mbatha, Mzubanzi Mabongo, David Owen Bates, Zodwa Dlamini

**Affiliations:** 1 SAMRC Precision Oncology Research Unit (PORU), Pan African Cancer Research Institute (PACRI), University of Pretoria, Hatfiel, Pretoria 0028, South Africa; rahaba.marima@up.ac.za (R.M.); flavia.zita4@gmail.com (F.Z.F.); rodney.hull@up.ac.za (R.H.); thulo.molefi@up.ac.za (T.M.); meryl.oyomno@up.ac.za (M.O.); richard.khanyile@up.ac.za (R.K.); sikhumbuzo.mbatha@up.ac.za (S.M.); mzubanzi.mabongo@up.ac.za (M.M.); David.Bates@nottingham.ac.uk (D.O.B.); 2 Department of Medical Oncology, Steve Biko Academic Hospital, University of Pretoria, Hatfield, Pretoria 0028, South Africa; 3 Department of Surgery, Steve Biko Academic Hospital, University of Pretoria, Hatfield, Pretoria 0028, South Africa; 4 Department of Maxillofacial and Oral Surgery, School of Dentistry, University of Pretoria, Hatfield, Pretoria 0028, South Africa; 5 Centre for Cancer Sciences, Division of Cancer and Stem Cells, Biodiscovery Institute, University of Nottingham, Nottingham NG7 2RD, UK

**Keywords:** MicroRNA (miRNA), alternative mRNA splicing (AS), drug resistance, chemotherapy

## Abstract

Cancer is a multifaceted disease that involves several molecular mechanisms including changes in gene expression. Two important processes altered in cancer that lead to changes in gene expression include altered microRNA (miRNA) expression and aberrant splicing events. MiRNAs are short non-coding RNAs that play a central role in regulating RNA silencing and gene expression. Alternative splicing increases the diversity of the proteome by producing several different spliced mRNAs from a single gene for translation. MiRNA expression and alternative splicing events are rigorously regulated processes. Dysregulation of miRNA and splicing events promote carcinogenesis and drug resistance in cancers including breast, cervical, prostate, colorectal, ovarian and leukemia. Alternative splicing may change the target mRNA 3′UTR binding site. This alteration can affect the produced protein and may ultimately affect the drug affinity of target proteins, eventually leading to drug resistance. Drug resistance can be caused by intrinsic and extrinsic factors. The interplay between miRNA and alternative splicing is largely due to splicing resulting in altered 3′UTR targeted binding of miRNAs. This can result in the altered targeting of these isoforms and altered drug targets and drug resistance. Furthermore, the increasing prevalence of cancer drug resistance poses a substantial challenge in the management of the disease. Henceforth, molecular alterations have become highly attractive drug targets to reverse the aberrant effects of miRNAs and splicing events that promote malignancy and drug resistance. While the miRNA–mRNA splicing interplay in cancer drug resistance remains largely to be elucidated, this review focuses on miRNA and alternative mRNA splicing (AS) events in breast, cervical, prostate, colorectal and ovarian cancer, as well as leukemia, and the role these events play in drug resistance. MiRNA induced cancer drug resistance; alternative mRNA splicing (AS) in cancer drug resistance; the interplay between AS and miRNA in chemoresistance will be discussed. Despite this great potential, the interplay between aberrant splicing events and miRNA is understudied but holds great potential in deciphering miRNA-mediated drug resistance.

## 1. Introduction

Cancers are biologically diverse and heterogenic and pose a major global challenge with increasing incidence, prevalence and mortality particularly in low resource countries [1,2,3]. Cancer drug resistance poses a major challenge in the treatment and management of the disease. This warrants urgent attention to, and targeting of, molecular alterations, such as miRNA regulation and alternative mRNA splicing, which hold potent therapeutic potential. The same type of numerous alterations in molecular mechanisms that can drive the malignant transformation of cells can also drive the development of drug resistance. Molecular events such as alterations in microRNA (miRNA) transcription and aberrant splicing events contribute to drug resistance in many cancers [1]. MiRNAs are short non-coding RNAs (ncRNAs) that function in the regulation of RNA silencing and gene expression [2]. MiRNAs are involved in diverse biological functions such as cell proliferation and differentiation, homeostasis, metabolism, apoptosis, cell death, and can play a role as either tumor suppressors or as oncogenes [3,4,5].

Alternative splicing (AS) contributes to several cellular functions by maintaining the diversity of the proteome, producing several differently spliced mRNA from a single gene for translation [6]. Like the expression of miRNAs, alternative splicing events are also rigorously regulated processes and aberrant splicing events give rise to malignancy and resistance to therapy [6]. Alternative splicing is known to take place in many genes that can promote or inhibit drug resistance. Cancer cells are known to make use of alternative splicing to alter their sensitivity to chemo-, radio-, hormonal, and immunotherapies by changing the expression profile of various protein isoforms. The changes in alternative splicing and the resulting change in protein expression may affect the effectiveness of various treatments by altering drug uptake and efflux, altering the target of the drug, altering the conversion of the drug to its active state or metabolites, increased drug sequestration, drug inactivation, altered apoptosis, altered DNA damage response (DDR), altered cellular communication and immune system evasion [7].

Evidence suggests that there is an interplay between miRNAs and alternative splicing events that alters drug resistance [1]. However, this interplay is underreported and forms a significant research gap. Dysregulation of miRNA and splicing events promote carcinogenesis and drug resistance in cancers including breast, cervical, prostate, colorectal, ovarian and leukemia [1,4,8]. The increasing prevalence of drug resistance in cancer poses a substantial challenge in the management of the disease. As a result, these molecular alterations are highly attractive drug targets to reverse the aberrant effects of miRNAs and splicing events that promote malignancy and drug resistance. This review will outline the involvement of miRNAs and splicing events that facilitate tumor progression and induce drug resistance in breast, cervical, prostate, colorectal, and ovarian cancers as well as leukemia. MiRNA-induced cancer drug resistance; alternative mRNA splicing (AS) in cancer drug resistance; and the interplay between AS and miRNA in chemoresistance will each be discussed.

## 2. Normal and Aberrant Biosynthesis of MiRNAs

**Figure 1:**
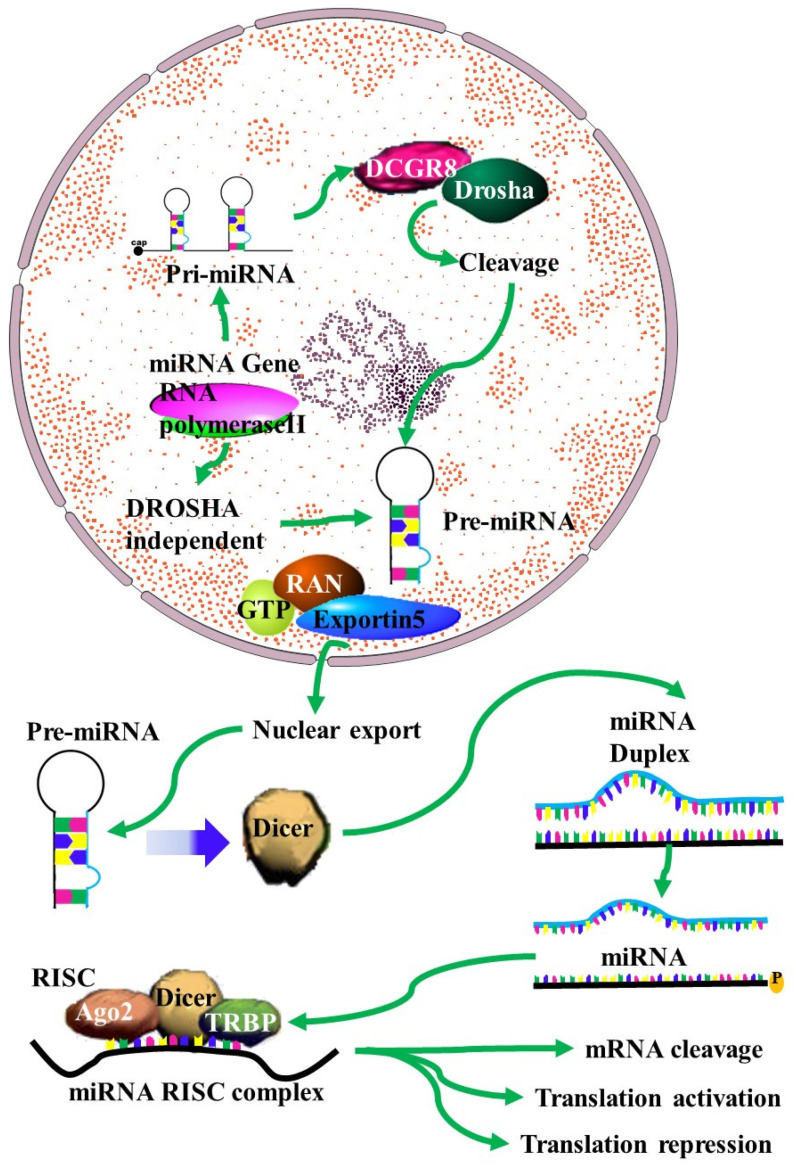
MiRNA biogenesis: A schematic outline of the biogenesis of miRNA. Following transcription by RNA polymerase II, the resulting primary microRNA precursor is then cleaved by the Drosha complex to generate pre-miRNA that is exported to the cytoplasm by Exportin-5. In the cytoplasm it is processed by Dicer into a miRNA duplex. The guide strand (mature miRNA) is then incorporated into the miRNA-induced silencing complex (miRISC) complex where gene silencing can be accomplished via mRNA target cleavage (degradation), or through the prevention of translation [4].

MiRNAs have been identified as vital components in carcinogenesis, including chemoresistance [9]. The mechanisms of action of miRNA in chemoresistance are emerging as crucial factors in anti-cancer therapeutic research. Despite this, these mechanisms remain to be fully understood. A single miRNA can act as a double-edged oncogenic sword, as either a tumor suppressor or an oncogene [10]. MiRNA can target genes related to drug targeting, transport and detoxification, cell cycle regulation, DNA repair and apoptosis (Figure 1) [1,11,12,13,14,15,16,17,18,19]. This process involves the transcription of miRNA transcripts to primary miRNA (pri-miRNA) in the nucleus by RNA pol II. This is followed by pri-miRNA processing to release the precursor miRNA (pre-mRNA) by the Drosha/DGCR8 complex. The released pre-miRNA is about 70nt in length and has a stem loop structure with a 2nt 3′ overhang [20]). The export of the pre-miRNA from the nucleus to the cytoplasm is facilitated by its binding to the Exp5/Ran-GTP complex. In the cytoplasm, the stem-loop pre-miRNA is processed into double-stranded RNA (dsRNA) by the Dicer/TRBP/PACT complex. The helicase enzyme then unwinds the ds-miRNA into single strands. Of the two miRNA strands, the strand with higher stability at the 5′ end will be degraded, while the strand with lower stability at the 5′ end will become a mature miRNA by integrating into the RNA-induced silencing complex (RISC). The miRNA–RISC complex will then bind to the 3′UTR of the target mRNA, inhibiting its translation [20].

Changes in the levels of miRNA in cancer are largely due to changes in the miRNA biosynthesis pathway. One of the causes of this is changes in the expression of proteins involved in these pathways. For instance, both DROSHA and DICER are downregulated in many cancers [21,22]. However, different oncoproteins have different effects on *DROCHA* transcription. It is downregulated by ADARB1 [23] but upregulated by MYC [24]. This leads to increased miRNA expression in some cancers and decreased miRNA expression in others. DICER levels in cancer are regulated in part by changes in the transcription factor Tap63, which is responsible for *DICER* transcription [25]. Mutations in p53 can also lead to a decrease in *DICER* levels [26], while *DICER* mRNA is also a target of miRNAs whose expression changes in many cancers [26].

Other than changes in biogenesis, miRNA expression profiles can change as a result of DNA damage response pathways altering the phosphorylation of KH-type splicing regulatory protein. Since some miRNAs can be generated from excised RNA originating from alternate splicing, this can change the expression levels of these miRNAs [27]. Additionally, the export of precursor miRNA to the cytoplasm can result from mutations or altered transcription of the *XPO5* gene [28].

## 3. Normal and Aberrant mRNA Splicing Events: Targeting the 3′UTR

### 3.1. Normal and Aberrant Splicing Events in Cancer

Alternatively spliced mRNA transcripts encode structurally, and perhaps functionally, distinct protein isoforms by exon retention or exclusion. More than 95% of human genes undergo AS. On average human genes have about 8 to 10 exons separated by the non-coding intronic regions, which can be 10 to 100 times longer [6]. In human cells, different mechanistic types of AS exist and these include: (1) intron retention in the mature mRNA transcript; (2) exon skipping where the whole exon is excluded from the mature mRNA transcript, (3) alternative 5′ splice site, with an alternative selection of 5′ splice sites, (4) alternative 3′ splice site, where there is an alternative selection of 3′ splice site. Alternative 5′ or 3′ splice site selection may lead to smaller exon retention, (5) mutually exclusive exons, where distinct exon combinations are generated, (6) alternative promoter selection, where alternative 5′ ends are generated by different pol II promoter selection, (7) alternative polyadenylation sites, where alternative 3′ ends are generated by the selection of different polyadenylation sites [6]. Alternative mRNA splicing is important in normal physiology, but aberrant splicing events have been reported in regard to cancer and drug resistance. It has been reported that aberrant splicing events result in novel mRNA transcripts not observed in normal cells. Furthermore, these novel mRNA transcripts have been reported in cancer and drug resistance [29]. Changes in alternate splicing that occur as a result of cancer largely occur due to genomic splice site point mutations. For instance, multiple splice-site mutations occur in p53 in various types of cancer. Exons are normally flanked by 5′ and 3′ intronic dinucleotides and mutations in these splice sites can lead to exon exclusion [30], or even double exon skipping [31]. Another common mechanism whereby mutations that arise during cancer give rise to aberrant splicing is through the creation of cryptic splice sites. This can lead to the inclusion or exclusion of nucleotides from an mRNA [32]. Cancer has also been observed to have higher rates of mutation in exonic splicing enhancers. Splicing factors bind to these sequences and recruit the spliceosome to splice sites which would normally not be recognized as splice sites. These mutations are known to disrupt the binding of splicing factors such as SC35 and ASF/SF2 [33,34,35].

Mutations in the genes that code for those aforementioned splicing factors that control and regulate splicing also regularly occur in cancers, resulting in changes in alternate splicing. For instance, mutations in the *SF3B1* gene are clustered in multiple hotspots. These mutations are all clustered within the Huntingtin, Elongation factor 3, protein phosphatase 2A (HEAT) domain, which is normally associated with intracellular transport [36]. Altered availability of this protein in the cytoplasm may explain the effect these mutations have on the function of these proteins. Mutations in the *SRSF2* splicing factor mostly consist of insertions/deletions in a hotspot around residue P95. This region occurs in a linker sequence between the N-terminal RNA recognition motif (RRM) and the C-terminal RS domain. The effect of these mutations is largely negative, being associated with decreased patient survival and poorer treatment outcomes [37]. Mutations in the zinc finger RNA binding motif and serine rich 2 (ZRSR2) splicing factor do not occur in a hotspot and are found across the gene sequence. These mutations mostly lead to reading frame changes and mutant proteins with decreased or no function. This results in altered splicing of some proteins which can best be described as U12-type intron retention [38]. Mutations in the zinc finger RNA binding motif and serine rich 2 (ZRSR2) splicing factor do not occur in a hotspot and are found across the gene sequence. These mutations mostly lead to reading frame changes and mutant proteins with decreased or no function. This results in altered splicing of some protein which can best be described as U12-type intron retention [38].

This results in protein isoforms that may lack full activity, have no activity, or even have the opposite activity. The expression of these isoforms can therefore result in the decreased activity of proteins that function as tumor suppressor genes.

### 3.2. Targeting the 3′UTR of Different Transcripts Generated by Alternative Splicing

**Figure 2:**
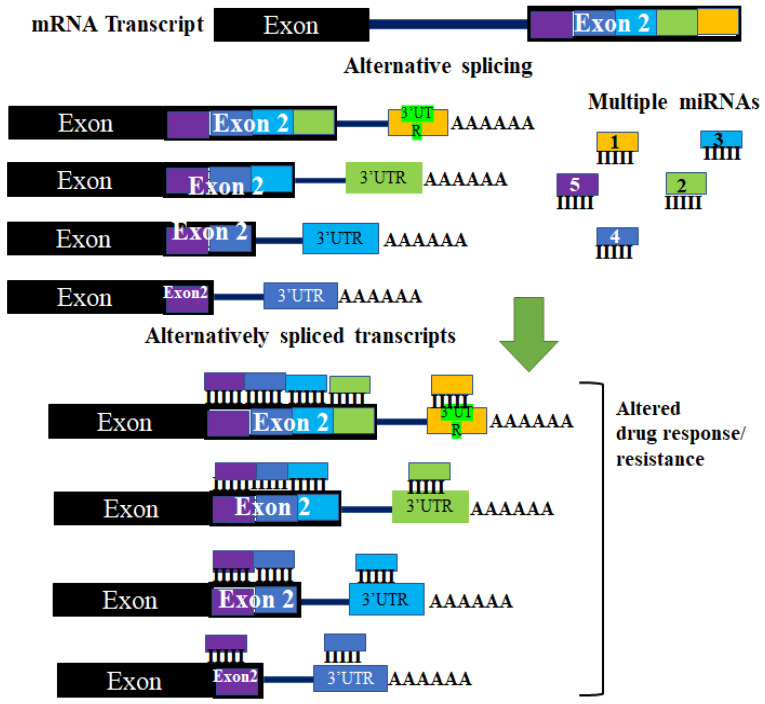
The effect of aberrant alternative splicing in miRNA regulation. Changes in the 3′UTR of target mRNA occur due to different polyadenylation sites. The different 3′ UTRs that result have different regions where miRNAs can bind and target the mRNA. These changes can result in some miRNAs not being able to regulate different alternatively spliced transcripts of the same pre-mRNA. These changes can affect regulation by miRNAs. This may lead to changes in cellular effectors (such as proteins and enzymes) of drug resistance, thereby altering a tumor’s response to a drug.

The ability of miRNAs to bind and regulate mRNA predominantly occurs at the 3′ UTR of the mRNA. Although occasionally this can occur at the 5′ UTR of the target mRNA [39,40]. Several studies have shown that introns retained at the 3′ UTR following alternative splicing can increase the number of putative miRNA target sequences [41], while truncated 3′ UTRs transcripts can alter miRNA binding affinity to targeted mRNA (Figure 2) [42]. The generation of alternative 3′ UTRs occurs through the alternative polyadenylation sites splicing mechanism. The patterns and occurrence of multiple alternative polyadenylation (APA) sites are known to be altered in cancer cells [43]. Ceramide is important for multiple physiological roles and is involved in the actions of some chemotherapy drugs. As a result of this, the downregulation of ceramide synthetase (CerS) is important in acquired drug resistance and metastasis. CerS is downregulated through the interaction between histone deacetylases (HDACs) and miRNAs. In addition to this, an isoform of CerS, CerS1, is detected at higher levels in cancer cells and the CerS1 spliced isoform is targeted for degradation by miR-574-5p by binding to the 3′ UTR within the retained intron between exons 6 and 7 [44].

## 4. MiRNA and Alternative Splicing-Induced Drug Resistance

### 4.1. Breast Cancer

Breast cancer poses a significant challenge to women globally and has a high mortality rate in low- and middle-income countries (LMICs) [45]. LMICs are particularly vulnerable to the effects of breast cancer due to the lack of early detection resulting in later stage cancers being diagnosed, which require more aggressive chemotherapy for which there are insufficient adequate treatment options [46]. Paclitaxel (PTX), cyclophosphamide (CYA), methotrexate, 5-fluorouracil (5-FU), and doxorubicin/Adriamycin (DOX) are commonly used chemotherapy drugs to manage breast cancer [47,48]. However, treatment failure is commonly observed in patients who tend to develop resistance to these drugs, which is attributed to miRNA modifications and aberrant alternative splicing.

#### 4.1.1. Alternative Splicing and Drug Resistance in Breast Cancer

Many of the mechanisms whereby alternative splicing influences drug resistance in breast cancer involve changes in the expression or activity of splicing factors. This then changes the protein isoform expression profile. Platinum-based compounds such as cisplatin are commonly used to treat breast cancer. Serine-arginine protein kinase 1 (SRPK1), is acetylated by the histone acetyltransferase Tip60. This results in a decrease in the kinase activity of SRPK1, changes in the stability of this protein and, therefore, changes in its ability to regulate alternative splicing. This increase in the acetylation of SRPK1 is accompanied by an increase in the sensitivity of breast cancers to cisplatin [49]. Doxorubicin is a widely used chemotherapeutic agent for the treatment of breast cancer. Multiple splicing events and splicing factors have been identified to be involved in doxorubicin resistance. Splicing events are controlled by the splicing factors Zinc finger Ran-binding domain-containing protein 2 (ZRANB2) and the splicing factor (SYF2). The activities of these splicing factors have been found to show an increase in doxorubicin resistance. One of the targets of these splicing factors is the transforming protein ECT2. These splicing factors lead to the increased expression of an ECT2 splice variant which includes exon 5 (ECT2-Ex5+). Doxorubicin-resistant cells accumulated in the S-phase following doxorubicin treatment and demonstrated increased expression of ECT2-Ex5+ [50]. Furthermore, it has been shown that hotspot mutations in spliceosome factor 3B1 (SF3B1) and upregulation of SF3B3 and serine arginine splicing factor 1 (SRSF1) result in resistance to endocrine therapy in breast cancer. The changes in the activity of these splicing factors results in changes in the expression of growth factor receptor proteins such as HER2. The d16HER2 splice variant of this protein prevents the therapeutic antibody Trastuzumab from blocking this receptor, leading to increased proliferation and resistance to this therapy [51]. This variant lacks exon 16 and the C terminal region and therefore has no tyrosine kinase domain. Another HER2 variant, the p100 variant, also reduces Trastuzumab efficacy [52].

#### 4.1.2. MiRNA-Induced Drug Resistance in Breast Cancer

Several miRNAs are implicated in drug resistance by interfering with regulatory and DNA damage repair pathways. For instance, Ren et al. (2017) showed how the interaction between miR-206 and WW domain binding protein 2 (WBP2) in breast cancer could alter tamoxifen resistance [53]. WBP2 has oncogenic potential, and its overexpression is observed in estrogen receptor positive (ER+) breast cancer where it promotes proliferation and facilitates the development of malignancy by activating oncogenes [54]. The authors showed that knockdown of WBP2 and overexpression of miR-206 rendered breast cancer cells less resistant to tamoxifen [53]. This suggests that the mechanism behind tamoxifen resistance in breast cancer cells can be deciphered by altering the association between WBP2 and miR-206. In addition, miR-26a is expressed at low levels in breast cancer compared with normal tissue and evidence shows that tamoxifen sensitivity is attained when miR-26a is expressed at high levels [55]. A study by Zhang et al. (2016) [56] shows that miR-365 is also expressed at low levels in breast cancer cells. This research showed that sensitivity to 5-FU can be attained by enhancing expression of miR-365 [56]. This miRNA targets TIF1, a regulator of ribosomal gene transcription [57]. Song et al. (2018) [58] showed similar results for miR-22 in breast cancer cells. Upregulating miR-22 in breast cancer cells overcame drug resistance and increased sensitivity to PTX [58]. This miRNA functions by altering estrogen signaling by directly targeting the estrogen receptor [59]. These results indicate the significance of miRNA in drug resistance in breast cancer.

Recent evidence shows that the enhanced expression of E2F transcription factor 7 (E2F7) and the inhibition of miR-26a in ER+ breast cancer confers resistance to tamoxifen, suggesting a feedback loop [60]. Silencing the feedback loop to enhance the expression of miR-26a and downregulation of E2F7 may promote tamoxifen sensitivity in breast cancer cells [60]. The target of miR-26a is the cell proliferation regulator CKS2 [61]. Additionally, E2F7 inhibits miR-15a/16 expression in breast cancer cells and the inhibition of these miRNAs is closely associated with tamoxifen resistance [62]. These miRNAs target another regulator of proliferation, HGF [63]. MiR-15a/16-related Tamoxifen resistance in breast cancer patients is induced by the inhibition of Cyclin 1 that promotes cell cycle arrest [64]. Drug resistance may be reversible by targeting these miRNAs or proteins that modulate the expression of these miRNAs. More recent evidence demonstrates that plasma miR-200a and miR-210 are elevated in metastatic breast cancer patients and are associated with chemotherapy resistance [65], while elevated levels of miR-210 is associated with Trastuzumab resistance in human epidermal growth factor receptor (HER)-positive breast cancer [64]. MiR-200a and miR-210 may also be valuable biomarkers as prognostic indicators of breast cancer stage and metastasis, respectively [65]. Both these miRNAs promote proliferation by inhibiting the ZED1 and ZEB2 transcriptional repressors [66] or the pro-apoptotic BCL11A [67]. Collectively, these results highlight that miRNA may be beneficial in predicting drug response in breast cancer patients and their significant role in inducing drug resistance. Table 1 shows alternative splicing events and miRNAs involved in breast cancer drug resistance.

### 4.2. Cervical Cancer

After breast cancer, gynecological cancers are the second most diagnosed cancers in women. Cervical cancer is the fourth most diagnosed cancer globally and a prominent challenge, particularly in developing countries such as those within the Sub-Saharan African (SSA) region, with 90% of cervical cancer-related mortality [45,86]. Cervical cancer is treated with surgery, radiotherapy, chemotherapy, or a combination of both radio- and chemotherapy. Cisplatin, paclitaxel, and carboplatin are used as frontline drugs for the management of cervical cancer. The response rate of cervical cancer patients to chemotherapy is 29–59% due to acquired chemoresistance [87,88].

#### 4.2.1. Alternative Splicing in Drug Resistance in Cervical Cancer

As in breast cancer one of the ways in which alternative splicing can contribute to drug resistance in cervical cancer is through changes in the splicing factors that regulate alternative splicing. The CRK-like (CRKL) adapter protein regulates alternative splicing of genes related to cancer. Some of the splice variants of these genes promote malignant transformation, metastases, and chemoresistance. These isoforms accomplish this by activating Src and Akt signaling pathways [74]. In the same way, they contribute to cisplatin resistance in cervical cancer [75].

#### 4.2.2. MiRNA-Induced Chemo and Radioresistance in Cervical Cancer

The association between miRNAs and drug resistance in cervical cancer patients has been investigated. In vitro evidence shows that cervical cancer cells with suppressed levels of miR-125a demonstrated resistance to paclitaxel treatment [89]. However, patients with enhanced levels of miR-125a typically had favorable outcomes in treatment and prognosis following treatment with a combination of paclitaxel and cisplatin. Inhibition of the miR-125a target, which is STAT3, promotes upregulation of miR-125a and thereby reversing paclitaxel drug resistance in cervical cancer cells [89].

Cervical cancer is frequently diagnosed at late-stage in LMIC, and radiotherapy remains the primary option for treatment for late-stage cancer, particularly in inoperable tumors [90]. An estimated 60% of cervical cancer patients receive radiotherapy as a standard treatment [91], yet patients acquire resistance to radiotherapy and this is the main contributing factor to treatment failure and recurrence [92]. Evidence shows that several miRNAs are closely correlated with radio resistance in cervical cancer. Genomic studies conducted on cervical cancer cells have elucidated miRNA signatures that promote radio resistance. The study by Zhang et al. (2013) [93] showed 14 miRNAs (miR-1246, 1290, 137, 150*, 3138, 3663-3p, 371-5p, 3926, 4271, 4327, 572, 584, 630, 765) were upregulated in cervical cancer cell lines and 6 miRNAs (miR-BHRF1-1, 1271, 15b*, 19b-1*, 378*, 95) were downregulated in radioresistant cervical cancer cell lines [93]. The authors identified a signature of upregulated miRNAs (miR-630, 1246, 1290, 3138) that is associated with enhanced radio resistance in cervical cancer [93]. MiR-630 targets insulin like growth factor receptor, regulating cancer progression and the sensitivity of cancers to drugs that target HER2 [94]. Other miRNAs altered in cervical cancer include miR-125a [95] and miR-320 [96] that are downregulated in radioresistant cervical cancer cells. The expression levels of these miRNAs determine the radio-phenotype of cervical cancer cells. An effective response to radiotherapy can be achieved by modulating the targets of miR-125a and miR-320, which are CDKN1A and β-catenin, respectively [95,96]. By altering the expression of this miRNA signature, radio resistance may be attenuated. A summary of the roles played by alternative splicing events as well as miRNAs is given in Table 2.

### 4.3. Prostate Cancer

Most prostate cancer patients are treated with anti-androgen therapy at the initial diagnosis. However, most patients ultimately develop resistance to this anti-androgen therapy [3,4]. Following lung cancer, prostate cancer is a second leading cancer in men globally. Developed countries have higher prostate cancer prevalence (70 per 100,000) compared with low to middle-income countries (LMICs). Contrarily, developed countries have lower prostate cancer mortality (10 per 100,000) rates compared with LMICs [100].

#### 4.3.1. Alternative Splicing in Prostate Cancer

The number of splicing events was found to be elevated in prostate cancer, correlating with the aggressiveness of the cancer but correlating inversely with the degree of differentiation of the cancer. Prostate cancer is often treated using androgen deprivation therapy (ADT), also known as castration therapy. Tumors resistant to this are known as castration resistant PCa (CRPC) and are treated with androgen receptor (AR) blockers, such as Enzalutamide (Enza). Alternative splicing of the androgen receptor has been observed in CRPC [101]. An analysis of splicing events in drug resistant prostate cancer revealed alternative splicing events in RNA binding proteins that are under control of the Myc signaling pathway. Many of these splicing events involve frameshifts or premature stop codons [102]. AR-V7 is an AR splice variant that lacks a ligand binding domain, resulting in the inability of this isoform to be blocked by drugs that bind to this receptor. Increased expression of this isoform leads to increased drug resistance. The splicing of AR that gives rise to AR-V7 is under the control of the RBM39 splicing factor. The anti-cancer drug indisulam promotes the degradation of RBM39, decreasing the expression of AR-V7 [103]. Splicing of AR-V7 is also controlled by nuclear ribonucleoprotein L (HNRNPL) HNRNPA1 and HNRNPH [104]. Additionally, the SF3B2 splicing factor promotes the expression of AR-V7 and consequently higher expression levels of this splicing factor is associated with an aggressive PCa phenotype [105].

#### 4.3.2. MiRNA-Induced Drug Resistance in Prostate Cancer

Aberrant miRNA expression has also been reported in prostate cancer. For example, MiRNA-148a was reported to be downregulated in hormone-resistant prostate cancer cells PC3 and DU145 compared with hormone-sensitive prostate cancer cells (LNCaP) and normal epithelial prostate cells PrEC. Transfection of prostate cancer cells with miRNA-148a precursor, sensitized these cells to anti-cancer drug PTX, inhibited cell migration and invasion and overall cell growth and proliferation. In this study, the mitogen and stress activated kinase 1 (MSK1) was identified as a direct target of miRNA-148a [106].

Furthermore, miRNA-143 was reported to downregulate KRAS in prostate cancer cells, resulting in inhibited proliferation and migration. Overexpression of this miRNA sensitized prostate cancer cells to docetaxel by targeting the EGFR/RAS/MAPK signaling pathway [107]. In addition, miRNA-34a expression has been reported to be decreased in prostate cancer cells, compared with normal prostate cells, while its normal expression controls the resistance of prostate cancer cells to PTX [108]. Downregulated expression of miRNA-34a confers PTX resistance by upregulating Sirtuin 1 (SIRT1), important in cellular response to inflammatory and oxidative stress and BCL-2 expression. These results indicate that miRNA-34a could be further targeted for drug resistance therapeutic purposes in hormone-resistant prostate cancer. In prostate cancer, miRNAs are reported to be modulators of androgen deprivation therapy (ADT) resistance [109]. Sun et al., (2012) [110] have reported that miRNA-221/222 are upregulated while miRNA-23b and miRNA-27b are downregulated in about 90% of metastatic castrate-resistant prostate cancer (mCRPC) cases compared with primary tumors. The mRNA target of miR-221/222 is the negative cell cycle regulator p27Kip1 miRNA-221/222, whose downregulation results in increased proliferation while the miRNA target of miRNA-23b is cyclin D1 [36] and functions to decrease proliferation. The role of alternative splicing events and miRNAs in resistance to drug treatment in prostate cancer (Table 3).

### 4.4. Ovarian Cancer

Ovarian cancer is reported to be the most lethal of the female gynecological cancers [4,112]. The first line of chemotherapeutic treatment is the CDDP/carboplatin combination with PTX, for advanced ovarian cancer. The role played by alternative splicing and miRNA in drug resistance in ovarian cancer is discussed below.

#### 4.4.1. Alternative Splicing in Drug Resistance in Ovarian Cancer

Extracellular matrix protein-1a (ECM1a) is a secreted isoform of ECM1. This isoform induces tumorigenesis by activating the AKT/FAK/Rho/cytoskeleton signaling pathway. In ovarian cancer, not only does this ECM isoform promote tumorigenesis but it also promotes cisplatin resistance by increasing the expression of CD326. The non-secretory isoform which inhibits the phosphorylation of myosin by binding to it, blocks cytoskeletal-induced tumorigenesis. ECM1b increases the expression of hnRNPLL splicing factor, this leads to increased splicing of ECM1 thereby generating multiple isoforms such as ECM1a [113].

#### 4.4.2. MiRNA-Induced Drug Resistance in Ovarian Cancer

MiRNA regulation in ovarian cancer drug response/resistance has also been studied. For example, miRNA regulation in ovarian cancer cells in response to CDDP is frequently studied. Such reports indicate that miRNAs such as miRNA-449a [114], let-7 [115], miRNA-130b [116], miRNA-370 [117], miRNA-199b-5p [118], miRNA-489 [119] and miRNA-9 [116] could all decrease the resistance of ovarian cancer cells to CDDP. These miRNA gene targets include cell cycle regulation genes, apoptosis, proliferation, angiogenesis-related genes, tumor suppressors and DNA repair genes [120,121]. Controversially, since one miRNA can target multiple genes regulating similar or contradicting pathways, miRNA-106a and miRNA-130a have been reported to play contradictory roles to CDDP sensitivity and drug resistance in ovarian cancer cells [98]. MiR 106a is thought to play an anti-proliferative role by targeting FOXC1 [122], while miR-130a suppresses cancer cell invasion and migration by targeting the FOSL1 transcription factor [123].

In addition, Boyerinas et al., (2012) demonstrated that the let-7 miRNA family targets the insulin-like growth factor (ILGF) mRNA-binding protein, which in turn destabilizes multidrug resistance 1 (MDR1) mRNA, thereby sensitizing ovarian cancer cells to taxol [115]. Furthermore, the miRNA-200 family has also been shown to regulate ovarian cancer cell resistance to taxanes. Taxanes bind to and inhibit the depolymerization of the β-tubulin microtubules subunit, thereby causing cell cycle arrest and apoptosis. Cochrane et al., (2009) [124] illustrated that miRNA-200c inhibits class III β-tubulin genes and targets and inhibits ZEB1 and ZEB2 to suppress epithelial-to-mesenchymal transition (EMT). Additionally, miRNA-200c has also been shown to increase PTX sensitivity in ovarian cancer cells [125]. Cittely et al. (2012) [126] also have also showed that miRNA-200c enhances taxane sensitivity in in vitro ovarian cancer models by targeting the TUBB3 gene. Furthermore, downregulated miRNA-200c levels are associated with poor prognosis in ovarian cancer, as a xenograft human ovarian cancer model with restored miRNA-200c illustrated decreased tumor formation and burden. A combination of miRNA-200c with a chemotherapeutic drug could be an effective treatment to prevent ovarian cancer metastasis and invasion [127]. In addition, miRNA-591 and miRNA-31 have also been shown to be involved in taxane resistance in ovarian cancer cells [128,129]. Mi-RNA-130b also regulates ovarian cancer cells’ resistance to CDDP and taxol. Furthermore, hypermethylation of miRNA-130b was found to be linked to ovarian cancer tumorigenesis and drug resistance, while miRNA-130b restoration sensitized ovarian cancer cells to taxol and CDDP, using colony-stimulating factor 1 (CSF1) as a direct target [130]. Despite this, miR-130b is known to target the miRNA for the tumor suppressor PTEN and promote cisplatin resistance in lung cancer [131]. The lack of effective biomarker screening and lack of early symptoms for ovarian cancer diagnosis is a major obstacle in successful ovarian cancer outcomes [112]. The role played by alternative splicing and miRNA in drug resistance in ovarian cancer is shown in Table 4.

### 4.5. Leukemia

Alternative splicing events and miRNAs contribute to drug resistance in leukemia in a variety of ways, discussed below.

#### 4.5.1. Alternative Splicing in Drug Resistance in Leukemia

Databases of transcriptome data from acute myeloid leukemia (AML) found that over 2000 genes are known to undergo alternative splicing. In addition to this, mutations were identified in SF3B1, SRSF2, and U2AF1. These splicing factors are associated with thousands of splicing events [140].

The breakpoint cluster region protein (BCR)-ABL1 tyrosine inhibitors, dasatinib and nilotinib are both used to treat AML. The oncoprotein cancerous inhibitor of protein phosphatase 2A (CIP2A) is alternatively spliced to give rise to multiple isoforms. One of these isoforms, known as novel CIP2A variant (NOCIVA) was isolated from chronic myeloid leukemia (CML) cohorts. Both CML and amyloid myeloid leukemia (AML) samples were found to contain high levels of NOCIVA and lower levels of CIP2A. The ratio of NOCIVA/CIP2A mRNA was found to be a good predictor of patient survival. High levels of NOCIVA are also associated with dasatinib and nilotinib resistance. CIP2A promotes cancer by inhibiting the tumor suppressor PP2A-B56a. The NOCIVA isoform contains exons 1 to 13 and retains the capacity to bind to B56a. However, while CIP2A is a cytoplasmic protein, NOCIVA is a nuclear protein [141].

The pyrimidine analog cytarabine is commonly used to treat AML and needs to be monophosphorylated by deoxycytidine kinase to form the active compound cytarabine-5’-triphosphate, which is the active metabolite being incorporated into DNA during DNA synthesis. Certain AML patients were found to be resistant to cytarabine. This was found to be due to the alternative splicing of deoxycytidine kinase (dCK). These splice variants lack exons 2–6, with some of these alternatively spliced transcripts not even being translated to proteins, resulting in inactive enzyme [142]. Furthermore, in AMLs, inhibition of serine/arginine-protein kinase 1 (SRPK1) by SRPK1 kinase inhibitor SPHINX31, led to a Bromodomain-containing protein 4 (BRD4) isoform switch, from the short to long and not full BRD4 mRNA length inhibition. This BRD4 isoform switch significantly reduced the survival and proliferation of AML cells [143]. All these isoforms retain the bromodomains but have truncated c termini.

#### 4.5.2. MiRNA-Induced Drug Resistance in Leukemia

MiRNAs have also been reported to play a significant role in leukemia drug resistance [4]. For example, Hershkovitz-Rokah et al. (2015) [144] showed that miRNA-30e was downregulated in chronic myeloid leukocyte (CML) patient samples and cell lines. Additionally, upregulated miRNA-30e expression sensitized K562 leukemia cells to imatinib and induced apoptosis, thereby suppressing proliferation. MiRNA-30e was also shown to downregulate ABL expression, acting as a tumor suppressor [144]. MiRNA-203 was also demonstrated to sensitize CML cells to imatinib. In this study, miRNA-203 was found to be expressed at lower levels in CML patients’ bone marrow, while miRNA-203 upregulation in combination with imatinib could promote apoptosis in CML cells [145]. This miRNA targets the de-ubiquitinating enzyme USP26, which is responsible for preventing the proteolytic degradation of onco-proteins [146]. Contrarily, oncogenic miRNA-486 promotes imatinib resistance in CML cells by targeting PTEN and FOXO1 tumor suppressors [147]. Additionally, miRNA-21, which targets *Bcl2* mRNA [148], was reported to be upregulated in daunorubicin (DNR)-resistant leukemia cells, while miRNA-21 knockdown in these cells increased DNR cytotoxicity [149]. MiRNA-125b was also shown to be involved in DOX drug resistance in leukemia cells [150]. The role played by alternative splicing and miRNA in drug resistance in leukemia is shown in Table 5.

### 4.6. Colorectal Cancer

Colorectal cancer is the third most commonly diagnosed cancer in both males and females, and the second leading cause of cancer related mortality. The incidence and mortality rates of colorectal cancer are steadily on the rise, particularly in low- and middle-income countries (LMICs) [45,151]. For effective management of colon cancer, early diagnosis in combination with the option of surgery is essential and results in successful treatment outcome rates of 70–90% [152,153]. However, colorectal cancer is often diagnosed in advanced stages that are treated with 5-FU, leucovorin, oxaliplatin, irinotecan [154,155] and trifluridine [156]. Effective treatment response is suppressed in advanced metastatic colorectal cancers which is attributed to the rise in drug resistance [153,155]. The role of alternative splicing events and miRNAs in drug resistance in colorectal cancer is detailed in Table 6.

#### 4.6.1. Alternative Splicing Induced Drug Resistance in Colorectal Cancer

Colorectal cancer can be treated using antiangiogenic therapy. The expression of different isoforms of vascular endothelial growth factor A (VEGFA) influences the sensitivity or resistance of CRC to antiangiogenic therapy [157]. Since some of the isoforms of VEGFA are antiangiogenic isoforms, the alternative splicing that generates these isoforms gives rise to isoforms that have a completely antagonistic activity to the iconic VEGFA. One isoform, VEGFA_165_b was found to be a negative regulator of resistance to Bevacizumab. Bevacizumab is a monoclonal antibody that inhibits VEGF and angiogenesis [158]. VEDF_165_b lacks exon 6 and other isoforms also lack this exon which codes for a low complexity region of the protein which immediately follows the PDGF domain. The T-cell intracellular antigen (TIA-1) is a protein that processes VEGFA mRNA. The splicing of TIA-1 results in a truncated isoform (sTIA-1) [159]. Higher levels of full-length TIA-1 and lower levels of sTIA-1 result in increased expression of the anti-angiogenic VEGF isoform, VEGF-A_165_b [157]. Spleen tyrosine kinase (SYK) acts as a tumor suppressor protein that is alternatively spliced to give rise to a long (SYK(L)) and a short isoform (SYK(S)). Increased expression of the long form leads to decreased proliferation and metastasis, while these effects are not observed following increased expression of the short form. The increased expression of either of the isoforms leads to increased sensitivity to 5-fluorouracil [160].

#### 4.6.2. MiRNA-Induced Drug Resistance in Colorectal Cancer

Drug resistance in colorectal cancer is attributed to various molecular mechanisms such as reduction in drug uptake, enhanced efflux [161], inactivation of drugs, DNA damage, irregularities in the cell cycle checkpoint [162] and inhibition of apoptosis [163]. Recent evidence shows that several miRNAs play a significant role in inducing drug resistance in colorectal cancers. Zhang and Wang (2017) recently reviewed miRNA mediated drug resistance in colorectal cancer [5]. MiR-297, miR-451, miR-519c, miR-222, miR-1915 and miR-153 are all associated with the induction of multidrug resistance in colorectal cancer. The drug resistance is induced by or mediated by altered expression of several molecular targets such as effectors of important pathways such as cell cycle regulation [5]. MiRNA-altered pathways in colorectal cancer that give rise to drug resistance include the PI3K/AKT signaling pathway, Wnt/β-catenin signaling pathway and the TGF-β signaling pathway [164].

**Table 6:** Alternative splicing events and miRNAs involved in drug resistance in colorectal cancer cells.

Colorectal Cancer			
Alternative Splicing			
Splicing Event	Drug	Effect	Ref
Splicing of VEGFA	**Bevacizumab**	VEGFA_165_b binds to the antibody preventing it from binding to and blocking VEGF	[158]
TIA-1 spliced to truncated sTIA-1		Higher levels of full-length TIA-1 and lower levels of sTIA-1 increase the expression of VEGF-A_165_b (anti-angiogenic).	[159]
SYK is spliced to long (SYK(L) and short SYK(S)	**5-FU**	The increased expression of either of the isoforms leads to increased sensitivity to the drug	[160]
**miRNA**			
**miRNA**	**Drug**	**Effects**	**Ref**
miR-153	**Multidrug**	Alteration of the expression of multiple isoforms of specific protein targets. Many of these targets are involved in roles such as cell cycle regulation and cell death	[5]
miR-297			
miR-451			
miR-222			
miR-1915			
miR-10b	**5-FU**	Increased resistance to the drug by acting on BIM	[165]
miR-21		Induces resistance to the drug by downregulating hMSH2 expression	[166]
miR-23a		miR-23a enhances sensitivity to the drug by acting through the APAF-1/caspase 9 apoptosis pathway	[167]
miR-34a		miR-34a acts on *c-kit* by downregulating it (*c-Kit*) and increasing sensitivity to the drug	[168]
miR-96		Expression of miR-96 decreased XIAP and p53 stability regulator UBE2N, increased apoptosis and increased sensitivity to the drug	[169]
miR-203		miR-203 targets and decreases expression of *TYMS*, increasing sensitivity to the drug.	[170]
miR-497		miR-497 decreases the expression of Smurf1 leading to increased drug sensitivity.	[171]
miR-587		Regulates PPP2R1B expression increasing resistance to the drug	[172]
miR-20a		miR-20a targets and downregulates *BNIP2*, increasing resistance to the drug	[173]
miR-20a	**Oxaliplatin**	miR-20a targets and downregulates *BNIP2*, increasing drug resistance	[173]
miR-203		miR-293 negatively regulates ATM increasing resistance to the drug	[174]
miR-503-5p		miR-503-5p targets PUMA leading to decreased expression of PUMA, leading to increased resistance to the drug	[175]
miR-1915		Inhibits BCL-2 leading to increased sensitivity to the drug	[176]
miR-20a		miR-20a targets *BNIP2* downregulating BNIP2 and increasing resistance to the drug	[173]
miR-451	**Irinotecan**	miR-451 downregulates MIF which downregulates its target COX-2 increasing sensitivity to the drug	[177]
miR-1915	**Doxorubicin**	Inhibits BCL-2 expression, increasing sensitivity to the drug	[176]

BNIP2—BCL2/adenovirus E1B 19 kDa protein-interacting protein; COX-2—cyclooxygenase-2; MIF-macrophage migration inhibitory factor; hMSH—human DNA MutS homolog 2; PPP2R1B—Protein Phosphatase 2 Scaffold Subunit A beta; PUMA—p53 up-regulated modulator of apoptosis; Smurf1—SMAD ubiquitination regulatory factor 1; 2SYK—Spleen tyrosine kinase; TIA-1—T-cell intracellular antigen; VEGFA—vascular endothelial growth factor A; XIAP—X-linked inhibitor of apoptosis protein.

## 5. The Interplay between Alternative Splicing and MiRNA in Drug Resistance

### 5.1. mRNA AS/miRNA Interplay

The interplay between aberrant splicing and miRNA dysregulation is a topic of interest and is gradually emerging as an important target for research with the final aim of advancing cancer treatment [178]. For researchers to be able to assess how splicing modulation affects miRNA profiles, bioinformatics detection tools for alternative splicing such as mixture of isoforms (MISO) [179], replicate multivariate analysis of transcript splicing (Rmats) [180] and SUPPA2 [181] can detect differential splicing in various conditions. Furthermore, splicing motif analysis tools such as RNAcontext [182] and multiple expectation maximizations for motif elicitation (MEME) [183] can identify regulators of alternatively spliced intersections. The miRNA targets of splicing modulation can be detected using miRTarBase [184].

Alternative splicing increases protein diversity and this may affect important genes related to pro-drug activation and drug metabolism [185]. Modulations of proteome diversity caused by both alternative splicing and miRNA regulation may affect patients’ drug metabolism and response. These alterations also affect personalized medicine and drug design. Changes in the transcription levels or sequence of an miRNA as well as changes in the sequences targeted by miRNA, will affect the levels of the corresponding cellular effectors such as proteins or enzymes, leading to differences in patients’ pharmacodynamics and pharmacokinetics. Additionally, depending on the splice variant of a specific cellular effector (protein/enzyme) related to the metabolism of specific drugs, patients will have a poorer or improved response to therapies. Changes in splice variants and miRNA regulation affect gene regulatory networks in cells. It is these changes in mechanisms that will provide key patient information for personalized medicine, so that the efficacy of specific drugs being used to treat specific patients can be improved. Continuous generation of information related to variations in alternative spliced variants and miRNA regulation will aid in better understanding variations in gene regulatory networks associated with drug metabolism and resistance.

The ability of miRNAs to influence alternative splicing events can occur in a myriad of ways. Some miRNAs are able to act as natural antisense transcripts (NATs). NATs are miRNAs that can bind to and interact with cis-acting elements in pre-mRNAs affecting splicing. These miRNAs accomplish this through the altered selection of splice sites and the recruitment of different splicing factors. These miRNAs can be classified as either a cis-NAT or a trans-NAT, depending on where the miRNA is transcribed from. If it is transcribed from the complementary strand of DNA coding for the target mRNA, it is a cis-NAT. If the miRNA is transcribed from a different locus than the target mRNA then it is a trans-NAT [186]. Apart from targeting RNAs for degradation they also act to stabilize mRNAs or promote translation by binding to the mRNA 5′ UTRs [187]. Alternative splicing can also be regulated by miRNAs through the ability of some miRNAs to alter chromatin structure which will influence the recruitment of splicing factors. This will affect their interaction with pre-mRNAs. MiRNAs can also affect transcription by interacting with Polymerase III [187] and regulating histone modification and DNA methylation.

### 5.2. mRNA AS/miRNA Interplay in Chemoresistance: Targeting the ceRNA Networks

The miRNA recognition elements (MREs) are short regions of the mRNA located in the 3′ UTR. Different mRNAs have similar MREs and are targeted by one miRNA. Additionally, one mRNA may be targeted by various miRNAs due to multiple distinct MREs [173,174]. Interestingly, other non-coding RNAs such as the lncRNAs with similar MREs can also compete with mRNAs for miRNA binding. These competing events are fundamental to establishing the principle of competing endogenous RNAs (ceRNAs). Although this review focuses on miRNA/AS interaction, emerging evidence shows the active involvement of the ceRNA (lncRNA-miRNA-mRNA) network in chemotherapeutic drug resistance [175,176]. While the mRNA is the ultimate target to be regulated by the ceRNA network, two possible mechanisms employed by this (ceRNA) network in chemoresistance have been reported. The ceRNA network target, the mRNA, may act either as a promoter (first mechanism) or as an inhibitor (second mechanism) of drug resistance [177]. In the first mechanism, miRNA can bind to the MRE of the mRNA promoting drug resistance, thereby degrading, or inhibiting, its translation. In the second mechanism, the anti-oncogenic drug resistance inhibitor mRNA is downregulated or degraded by higher levels of miRNA expression leading to higher levels of binding to the mRNA MRE, thereby inhibiting its protein expression. In both mechanisms, the miRNA/mRNA interplay is crucial in chemoresistance, by either inhibiting or promoting chemoresistance. In the latter mechanism, the ceRNA network components such as the lncRNA with similar MRE may act as decoys, competing for the miRNA binding and possibly protecting the target drug resistance inhibitor mRNA from degradation [178,179]. Furthermore, there is an urgent need to complement the rich data of miRNAs’ role in drug resistance with the miRNA/AS interplay in different cancers including breast, cervical, prostate, colorectal, and ovarian cancers, as well as leukemia. Notably, although miRNA/mRNA is key to chemoresistance, the ceRNA network is fundamental to this interplay.

### 5.3. The Interaction between MiRNAs and Splicing Factors

The expression of splicing factors can be controlled by miRNAs in the same way they control the stability and translation of any mRNA, by binding to complementary sequences on the splicing factor mRNA which lead to its degradation or the downregulation of mRNA translation [188]. Multiple miRNA-related effects on splicing factors have been identified in many cancers (Table 7). This involves the regulation of multiple splicing factors through the action of miRNAs. For instance, SRSF6 is downregulated by miR-193a-5p leading to increased metastasis [189]. The HnRNP splicing factors are known to interact with miRNAs in facilitating the ability of cancer cells to resist docetaxel in ovarian cancer. Here, HnRNP1A1 suppresses miR-18a expression. This miRNA is known to downregulate KRAS expression, and the upregulation of KRAS following the downregulation of miR-18a facilitates resistance to docetaxel. HnRNPA1 expression has also been downregulated by the tumor suppressor miRNAs miR-15a-5p and miR-25-3p [190].

Additionally, many miRNAs require the action of splicing factors to be correctly produced. The miR-7 family requires the activity of SRSF1 to be correctly processed into mature miRNA but in an example of negative feedback, miR-7 suppresses the expression of SRSF1 [191]. SRSF1 is also known to be regulated by miR-221, miR-222 and miR-17-92. SRSF1 is known to be involved in the expression of many cancer promoting genes as well as in the expression of pro-apoptotic isoforms of Bcl-x, RON, and MCL-1 [191]. The splicing factor PTBP1 is responsible for the increased expression of an isoform of PKM which is associated with a worse prognosis for patients. PTBP1 is downregulated by miR-124. The expression of this miRNA results in the increased expression of the PKM1 isoform that suppresses cancer progression [192]. SF3B1 and Rbfox2 are other splicing factors that play a role in the maturation of MiRNAs. SF3B is involved in the maturation of miRNAs that include miR-103a miR-423, and multiple miRNAs that target overlapping sites on target miRNAs [193]. The RNA-binding Fox protein 2 (Rbfox2) is downregulated in many forms of cancer where it upregulates tumor suppressor miRNAs. This is surprising as Rbfox2 promotes cell invasion [194].

Retinoic acid has been studied as a treatment for lung cancer where it blocks invasion and metastasis [195]. It also reduces chemotherapy-induced neuropathy in an animal model [196]. The miRNAs, miR-10a and miR-10b are known to degrade SRSF1 mRNA in neuroblastoma. This results in change in the expression of certain isoforms due to altered splicing of some of the mRNAs that are targets for this splicing factor. This results in decreased migration, invasion, and metastasis of neuroblastoma cells. The expression of these miRNAs increases the sensitivity of cancer cells to retinoic acid [197].

**Table 7:** The interaction between miRNAs and splicing factors in drug resistance.

miRNA	Splicing Factor	Type of Alteration	Cancer	Effect Produced	Ref
miR-18a	**HnRNP1A1**	HnRNP1A1 suppresses miR-18a	Prostate	Docetaxel resistance. KRAS upregulation	[190]
miR-15a-5p		HnRNPA1 downregulated	Pancreatic	Tumor suppression	[190]
miR-25-3p					[190]
miR-7 family	**SRSF1**	Correct synthesis of miR-7 family	Pancreatic	SRSF1 is involved in the expression of many cancer promoting genes as well as in the expression of pro-apoptotic isoforms of Bcl-x, RON, and MCL-1	[191]
miR-7 family		miR-7 suppresses the expression of SRSF1	Prostate		[191]
miR-222		Downregulation of SRSF1	Prostate		[191]
miR-221			Pancreatic		[191]
miR-17-92			Pancreatic		[191]
miR-10a			Lung	Increased retinoic acid sensitivity	[197]
miR-10b			Neuroblastoma		[197]
miR-193a	**SRSF6**	Downregulation of SRSF1	Pancreatic	increased metastasis	[189]
miR-124	**PTBP1**	Downregulates PTBP1	Pancreatic	increased expression of an isoform of PKM worse prognosis	[192]
Tumor suppressor miRNAs	**Rbfox2**	Rbfox2 is downregulated in cancer	Multiple	Rbfox2 promotes cell invasion	[194]
miR-103a	**SF3B1**	SF3B1 regulates maturation of miR-103a	Pancreatic	Promotes tumor growth	[193]
miR-423		SF3B1 regulates maturation of miR-423	Ovarian	Inhibits proliferation and metastasis	[193]
miR-193a-5p	**SRSF6**	Alternative splicing of OGDHL and ECM1	Pancreatic	promotes cell metastasis	[189]
miR-184	**AF1**	LncRNA UCA1 promotes proliferation by suppressing MiR-184	Oral squamous cell carcinoma	cisplatin resistance	[198]
miR-221	**Quaking (QKI)**	miR-221 targets the most abundant isoform of QKI and reduced QKI-5 levels	Colorectal	Increased resistance to cytotoxic agents	[199]

HnRNPA1-heterogeneous nuclear ribonucleoprotein1 A1; SRSF1- Serine/arginine-rich splicing factor 1; SRSF6- Serine/arginine-rich splicing factor 6; PTBP1- Polypyrimidine Tract Binding Protein 1; SF3B1- Splicing factor 3B subunit 1; AF1-activation function 1; SRSF1- Serine and Arginine Rich Splicing Factor 1; RON- recepteur d’origine nantais; MCL-1- Myeloid-cell leukemia 1; PKM-Pyruvate kinase M; Rbfox2- RNA Binding Fox-1 Homolog 2; OGDHL- Oxoglutarate Dehydrogenase L; EMC1- extracellular matrix protein 1; LncRNA- long non-coding RNA; UCA1- Urothelial Cancer Associated 1. QKI-Quaking.

## 6. Clinical Implications of Chemoresistance

Resistance to anticancer agents is a major stumbling block in the treatment of various cancers with systemic therapy. This resistance retards the therapeutic effect and utility of conventional chemotherapy and drugs that target specific molecules in treating many cancers [200,201]. The reduced therapeutic effect allows the cancer’s biological processes to continue unabated, resulting in increased tumor burden and eventual metastases [202]. Mechanisms resulting in resistance can either be at a pre- or post-tumor level. Pre-tumor processes limit the amount of drug reaching its target, while tumor level mechanisms allow the cancer to evade apoptosis [203]. Therapeutic resistance can either be primary, through tumor and/or patient factors or it can be secondary following exposure to treatment, which causes diverse intra-tumoral modifications [200,204].

The distinction between these two forms of resistance is purely academic, as considerable overlap exits in vivo. The occurrence of intrinsic tumor resistance, as described by Goldie and Coldman is influenced by patient factors relating to drug pharmacokinetics, in the same way that acquired resistance is impacted by primary resistance [202,203,205]. This interplay adds difficulty in applying resistance reversal strategies, with treatment toxicity and increasing resistance to multiple lines of therapy being the major hurdles. Since the discovery of drug resistance, multiple strategies have been employed to overcome it. In the days of conventional chemotherapy, alternative drug dosing, scheduling and multiagent regimens were the initial approaches used to combat resistance. The advent of companion diagnostics has allowed the development of therapies that target specific molecules and led us into the era of personalized oncology care [206,207,208,209].

Personalized oncology has revolutionized the treatment of cancer by treating a patient’s tumor based on its genetic profile. Cisplatin resistance in gastric cancer is known to be induced through the action of miR-193a-3p which acts on SRSF2, downregulating splicing events that are controlled by this splicing factor. The levels of this miRNA are upregulated in drug resistant gastric cancer. This has led to pilot studies where the levels of miR-193a are used as a prognostic marker in gastric cancer patients, following surgery. In this case following surgery it is common to treat the patient with cisplatin to help prevent cancer recurrence. The presence of miR-193-3p would act as an indicator that not only are there still cancer cells present but that they are resistant to cisplatin [210].

This approach has, however, failed to resolve the issue of drug resistance, as tumor heterogeneity and resistance escape mechanisms still obstruct the route to a cure or more effective treatment for cancer [211].

The activation of resistance escape mechanisms has led to the search for alternative solutions, such as synergistic target therapy combinations, natural products, biologics and nanotechnology and drug delivery systems [206,208]. The use of antisense oligonucleotides (ASOs) to target miRNAs can be a useful therapy to eliminate drug resistance caused by the action of miRNAs. In glioblastoma the occurrence of cisplatin resistance can be caused by the overexpression of miR-10b. This miRNA is able to interact and alter the splicing activity of multiple splicing factors. Mouse models of glioblastoma treated with a combination of miR-10b ASOs in combination with cisplatin, resulted in a delay in cancer progression and metastasis compared with only cisplatin controls [212]. The complexities of cancers and biological systems continues to complicate the reversal of drug resistance when it occurs. The exploration and discovery of new tumor targets prompts the development of novel therapies, with growing hope of overcoming the hallmarks of cancer and nearing the possibility of a cure [213,214,215].

## 7. Conclusions and Perspectives

Cancer drug resistance has been a challenging topic for decades in cancer biology. While intrinsic factors (such as target mutations) play a significant role, extrinsic factors (such as drug inactivation) are also considerable participants in this daunting process. Wherein the key role of miRNA in tumorigenesis is being extensively studied, this article discussed the vital role miRNAs play, together with alternative splicing events in anti-cancer drug resistance. MiRNAs have been reported to possess a double-edged oncogenic role, either as tumor suppressors or as oncogenes in tumorigenesis. Similarly, miRNAs can also promote or reduce cancer drug resistance. Moreover, a single miRNA can induce drug resistance in various cancer cells. Although progress is being made to repress cancer drug resistance, completely eliminating the problem of drug resistance in cancer is a long road that requires a comprehensive approach. It is this multifaceted nature of miRNAs that render them as suitable candidates in combating cancer drug resistance. Given the large number of variations in miRNA that are observed in various cancers, miRNAs serve as suitable and effective targets and biomarkers to predict therapeutic responses and altered drug resistance. Furthermore, aberrant splicing events and miRNA dysregulation in drug resistance are often individually studied. As alternative splicing can alter the 3′ UTR targeted binding site for miRNAs, this can also lead to the modulation of these protein targets and hence altered drug targets or altered enzymes that act on these drug targets or on the drugs themselves. This can then give rise to drug resistance.

**Figure 3:**
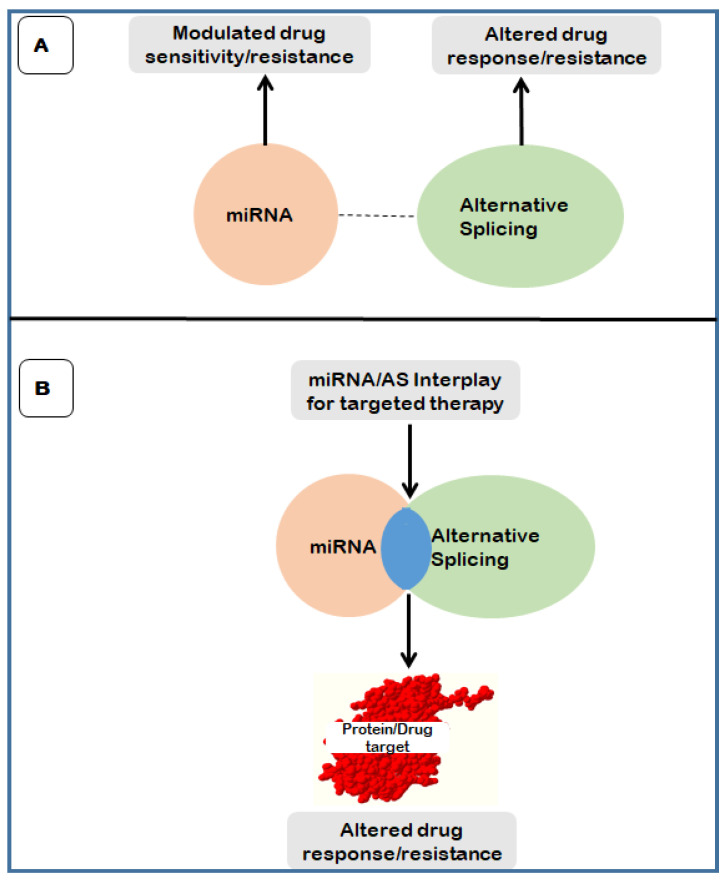
The potent miRNA/AS interplay target in anti-cancer drug resistance. Adequate research data is available on the individual roles of miRNA and AS in anti-cancer drug resistance (**A**). However, the research gap lies significantly in the interaction of these two strictly regulated processes (**B**), which holds potent therapeutic targets.

The interplay between these two stringently regulated processes in anti-cancer drug resistance is understudied and this further broadens the research gap of understanding and targeting this interplay in anti-cancer drug resistance. While adequate data is available on the role of each of these tightly regulated processes, current and future studies should focus on deciphering and targeting this interplay in anti-cancer drug resistance (Figure 3). Emerging research suggest that this interplay may be facilitated by ceRNA networks which include lncRNAs. The miRNA/mRNA interaction may act as inhibitors or promoters of anti-cancer drug resistance. Thus, miRNA/alternative splicing interaction holds the position of a potent target towards elucidating drug resistance. As alternative splicing can alter the 3′ UTR targeted binding of miRNAs, this can lead to modulated protein targets and hence altered drug targets which may result in drug resistance. The interplay between miRNA and alternative splicing also holds the promise of being a potent target for the development of biomarkers for the detection of underlying causes of drug resistance as well as the development and application of personalized medicine involving the use of specific drugs for the patient. This will involve using transcriptome sequencing to detect changes in miRNA and splice variants as indicators of which drugs may be effective and should be used to treat the patient. These signatures can also be used in the study and elucidation of the mechanisms behind drug resistance.

## Figures and Tables

**Table 1:** Alternative splicing events and miRNAs involved in drug resistance in breast cancer cells.

Breast			
Alternative Splicing			
Splicing Event	Drug	Effect	Ref
HER2 target of splicing factors resulting in increased expression of d16HER2 isoform	**Trastuzumab**	d16HER2 isoform stops trastuzumab blocking HER2 receptor, increasing proliferation	[52]
ECT2 spliced to form ECT2-Ex5+	**Doxorubicin**	Levels of splicing factors ZRANB2 and SYF2 is increased in resistant breast cancer. ECT2 is a splicing target of these splicing factors. Increasing levels of the ECT2-Ex5+ variant. This isoform increases resistance to the drug	[50]
SRPK1 and Tip60 are alternatively spliced	**Cisplatin**	Tip60 isoforms change in acetylation of SRPK1, changes in splicing activities.	[49]
**miRNA**			
**miRNA**	**Drug**	**Effect**	**Ref**
miR-22	**Paclitaxel**	Low levels pf miR-22 in breast cancer. High levels lead to increased drug sensitivity	[58]
miR-155	**PTX**	TGF-beta induces miR-155 expression, miR-155 reduced RhoA protein and disrupted tight junction promoting proliferation.	[68]
miR-365	**5-FU**	Low levels pf miR-365 in breast cancer. High levels lead to increased drug sensitivity	[56]
miR-125b		Expression pattern of miR-125 indicates its involvement with drug resistance through its effect on E2F3	[69]
mi-R206	**Tamoxifen**	Targets WBP2. Decreased expression of this oncogene leads to increasing drug sensitivity	[53]
miR-26a		Low levels pf miR-26a in breast cancer. High levels lead to increased drug sensitivity	[55]
		Negative feedback loop with E2F7 promotes sensitivity to treatment	[60]
miR-15a/16		E2F7 inhibits miR-15a/16 expression, increasing drug resistance, due to inhibition of Cyclin 1	[62,64]
miR-210	**Trastuzumab** **Tamoxifen**	Elevated levels of miR-210 is associated with drug resistance	[64]
Let-7b		Let-7b downregulates ER-α36 signaling leading to increased drug sensitivity	[70]
miR-200c	**Trastuzumab**	Targets ZNF217, ZEB1 resulting in suppressed TGFβ signaling, increasing sensitivity to the drug	[71]
miR-221		Targets PTEN for degradation, promoting drug resistance	[72]
miR-155	**VP-16**	TGF-beta induces miR-155 expression, miR-155 reduced *FOXO3A* and disrupted tight junction promoting proliferation	[68]
miR-30c	**Doxorubicin**	miR-30c targets *YWHAZ* for degradation, increasing resistance to the drug	[73]
miR-155		TGF-beta induces miR-155 expression, miR-155 reduced *Foxo1a* and disrupted tight junction promoting proliferation	[68]
miR-34a		miR-34a targets *Notch1* leading to decreased drug resistance	[74]
miR130b		miR-130b targets *PTEN* leading to increased resistance to the drug	[75]
miR-137		miR-137 targets *YB-1* leading to increased drug sensitivity.	[76]
miR-149		miR-149 targets *NDST1* leading to increased sensitivity to the drug	[77]
miR-195		miR-195 targets *RAF-1* leading to increased sensitivity to the drug	[78]
miR-200c		Mir-200c targets and decreases expression of *TrKB* and *BMI1*, leading to increased drug sensitivity	[79]
miR-298		miR-298 targets and decreases levels of *MDR1*, increasing drug resistance	[80]
miR-17 and miR-20b	**Taxol**	miR-17 and miR-20b targets and downregulates *NCOA3*, increasing sensitivity to the drug	[81]
miR-181a	**Adriamycin**	miR-181a targeted *BCL-2* leading to enhanced apoptosis and increased sensitivity to this pro-apoptotic drug	[82]
miR-34a	**Docetaxel**	miR-34a targets and decreases expression of *BCL-2* and *CCND1*, increasing drug resistance	[83]
miR-96	**Cisplatin**	miR-96 targets and downregulates the expression of *RAD51* and *REV1*, resulting in increased drug sensitivity	[84]
miR-218	**Taxol**	miR-218 targets *BIRC5* and decreases SURVIVIN-1 expression, leading to increased drug resistance	[85]
miR-20a	**Multidrug**	miR-20a targets *MAPK1* for degradation, inhibiting the MAPK1 signaling pathway downregulating the expression of P-gp and c-Myc, sensitizing cells to the drugs	[8]

BIRC5—Baculoviral IAP repeat-containing protein 5; CCND1—G1/S-specific cyclin; D1; E2F3—E2F Transcription Factor 3; ER-α36—Estrogen receptor alpha-36; FOXO1—Forkhead box protein O1; FOXO3a—Forkhead box protein O3;MAPK1—Mitogen-activated protein kinase 1; MDR1—Multidrug resistance protein 1; NCOA3—Nuclear receptor coactivator 3; NDST1—N-sulfotransferase 1; PTEN—Phosphatidylinositol 3,4,5-trisphosphate 3-phosphatase and dual-specificity protein phosphatase; REV1—DNA repair protein; Raf-1—RAF proto-oncogene serine, TGFβ—Transformation growth factor beta; TrKB—Neurotrophic tyrosine kinase receptor type 2; WBP2—WW domain binding protein 2; YB-1—Y-box-binding protein; YWHAZ—14-3-3 protein zeta/delta; ZNF217—Zinc finger protein 217; ZEB1—Zinc finger E-box-binding homeobox 1.

**Table 2:** Alternative splicing events and miRNAs involved in drug resistance in cervical cancer cells.

Cervical Cancer			
Alternative Splicing			
Splicing Event	Drug	Effect	Ref
CRKL regulates the splicing of genes related to cancer	**Multidrug**	Many mRNAs whose splicing is regulated by CRKL are related to malignant transformation, metastases, and chemoresistance	[97]
**miRNA**			
**miRNA**	**Drug**	**Effects**	**Ref**
miR-125a	**Paclitaxel**	Suppressed miR-125a shows increased resistance to the drug alone but increased sensitivity to paclitaxel and cisplatin combination. miR-125a targets *stat3*	[89]
miR-30a	**Cisplatin**	miR-27a suppresses Beclin1-induced autophagy and increases sensitivity to the drug	[98]
miR-214		miR-214 targets and downregulates *BCL2L2* expression and increases sensitivity to the drug	[99]

CRKL—CRK-like (Proto-oncogene).

**Table 3:** Alternative splicing events and miRNAs involved in drug resistance in prostate cancer cells.

Prostate Cancer			
Alternative Splicing			
Splicing Event	Drug	Effect	Ref
Alternative splicing of the androgen receptor (AR)	**Enzalutamide**	AR-V7 is an AR splice variant that lacks a ligand binding domain, resulting in increased resistance to the drug	[102]
**miRNA**			
**miRNA**	**Drug**	**Effect**	**Ref**
miR-143	**Docetaxel**	Downregulation of KRAS inhibits proliferation and migration. Increasing sensitivity to the drug by targeting EGFR/RAS/MAPK signaling	[107]
miR-200b		miR-200b enhances sensitivity to the drug by binding to and degrading *BMI1*	[111]
miR-148a	**Paclitaxel**	Increased levels of miR-148 led to increased sensitivity to the drug by targeting and decreasing expression of *MSK1*	[108]
miR-34a		miRNA-34a expression is decreased in prostate cancer. Decreased expression results in increased drug sensitivity due to upregulated SIRT1 and BCL-2 levels	[106]

**Table 4:** Alternative splicing events and miRNAs involved in drug resistance in ovarian cancer cells.

Ovarian Cancer			
Alternative Splicing			
Splicing Event	Drug	Effect	Ref
ECM1 is spliced to give rise to ECM1a	**Cisplatin**	ECM1 isoform induces tumorigenesis by activating the AKT/FAK/Rho/cytoskeleton signaling pathway and promotes resistance to the drug by increasing CD326	[113]
**miRNA**			
**miRNA**	**Drug**	**Effects**	**Ref**
Let-7b	**Tamoxifen**	Targets and downregulates *ER-α36*, increasing sensitivity to the drug	[70]
Let-7e	**Cisplatin**	Let-7e increased sensitivity to the drug by reducing the expression of proteins related to the increased resistance to the drug, namely BRCA1, EZH2, CCND1	[132]
miR-199b-5p		miR-199b-5p downregulated JAG1 leading to increased sensitivity to the drug	[118]
miR-93		Regulates PTEN/AKT signaling resulting in increased drug sensitivity	[121]
miR-106a		Increased resistance to the drug by targeting and decreasing PDCD4 expression	[133]
miR-130b		Increased sensitivity to the drug by targeting and downregulating CSF-1 expression	[134]
miR-214		Increases resistance to the drug by targeting *PTEN*	[135]
miR-370		miR-370 decreases the expression of ENG leading to increased drug sensitivity	[117]
miR-489		miR-489 increased sensitivity to the drug by targeting *Akt3*	[119]
miR-31	**Paclitaxel**	Increases MET expression leading to increased drug resistance	[128]
miR-136		miR-136 targets *Notch3* leading to increased sensitivity to the drug	[136]
miR-130b		Increased sensitivity to the drug by targeting and decreasing CSF-1 expression	[134]
miR-200c		miR-200c targets TuBB3, TrKB resulting in increased sensitivity to the drug	[126]
miR-145		miR-145 targets and downregulates expression of SP1 and CDK6, increasing the sensitivity to the drug	[137]
miR-591		miR-591 increases resistance to the drug by targeting *Bcl-10*, *Caspase7* and *Zeb1*.	[129]
miR-100	**Doxorubicin**	Re-sensitizes resistant cells to the drug by targeting *PLK1*	[138]
miR-197	**Taxol**	miR-197 regulates NLK expression to increase resistance to the drug	[139]
let-7		This miRNA family targets *IMP-1*, destabilizing MDR1 and sensitizing cells to the drug	[115]
miRNA-200c	**Taxane**	miRNA-200c inhibits class III β-tubulin by targeting *ZEB1* and *ZEB2*, increasing drug sensitivity	[124]
miRNA-200c		Targets *TUBB3* resulting in increased sensitivity to the drug	[127]

CSF1—colony-stimulating factor 1; ECM1a—Extracellular matrix protein-1a; ENG—Endoglin; EZH2—enhancer of zeste 2, ILGF—insulin-like growth factor (MDR1) multidrug resistance 1; NLK—Serine/threonine-protein kinase.

**Table 5:** Alternative splicing events and miRNAs involved in drug resistance in leukemia cells.

Leukemia			
Alternative Splicing			
Splicing Event	Drug	Effect	Ref
Cip2a alternatively spliced to form nociva	**Dasatinib and Nilotinib**	High levels of NOCIVA are also associated with dasatinib and nilotinib resistance	[141]
DCK splicing	**Cytarabine**	Some isoforms of DCK lack the ability to process cytarabine into its active metabolite, contributing to resistant AML	[142]
BRD4	**SPHINX31**	Short to long BRD4 isoform switch leads to reduced AML cell survival/proliferation and increased drug sensitivity	[143]
**miRNA**			
**miRNA**	**Drug**	**Effects**	**Ref**
miR-30e	**Imatinib**	Downregulated in *CML*. Increased miR-30e led to increased drug sensitivity via BCR-ABL expression	[144]
miR-203		Increased sensitivity to the drug by downregulating BCR-ABL expression	[145]
miR-486		Promotes resistance to the drug by targeting *PTEN* and *FOXO1*	[147]
miR-125b	**Doxorubicin**	Represses BAK1 protein expression leading to increased drug resistance	[150]

AML—acute myeloid leukemia; CIP2A—cancerous inhibitor of protein phosphatase 2A; CML—chronic myeloid leukocyte; DCK—deoxycytidine kinase; NOCIVA—novel CIP2A variant.
